# A Primary Liver Cancer Patient Treated With Stereotactic Body Radiation Therapy Using Diaphragm Motion Surrogate Tracking in CyberKnife

**DOI:** 10.7759/cureus.86463

**Published:** 2025-06-20

**Authors:** Shuiwang Qing, Chunshan Yu, Lei Gu, Yangsen Cao

**Affiliations:** 1 Department of Radiation Oncology, The First Affiliated Hospital of Naval Military Medical University, Shanghai, CHN

**Keywords:** diaphragm motion surrogate tracking, “liver cancer”, radiotherapy (rt), sbrt (stereotactic body radiotherapy), synchrony technique

## Abstract

Stereotactic body radiotherapy (SBRT) is one of the safe and effective treatment options for primary liver cancer. This case report describes a 64-year-old patient diagnosed with primary liver cancer after a contrast-enhanced T1-weighted MRI scan, which revealed a 4.4×5.0 cm mass in the upper segment of the right anterior lobe of the liver. The patient underwent SBRT using diaphragm motion surrogate tracking (DMST) with a dose of 45 Gy delivered in six fractions to the lesion, followed by oral Sorafenib and regular imaging follow-ups. Post-treatment MRI showed that the tumor had shrunk to 0.8cm. This case highlights the efficacy of CyberKnife SBRT in managing liver tumors near the diaphragm dome, achieving a radiographic response.

CyberKnife SBRT delivers high-dose radiation to tumors while sparing surrounding normal tissues through high-precision radiotherapy and tracking technologies, particularly suitable for respiratory motion-related lesions. In this case, the patient had a local progression at 23 months and died at 46 months after SBRT. This preliminarily shows the feasibility of integrating DMST into the respiratory motion model for CyberKnife SBRT in treating liver tumors adherent to the diaphragm dome.

## Introduction

Liver cancer is one of the most common malignancies globally, with hepatocellular carcinoma (HCC) accounting for over 90% of primary liver cancers [1]. HCC typically arises from chronic liver diseases such as alcoholic cirrhosis, chronic viral hepatitis, and metabolic disorders [2]. Treatment decisions for HCC are primarily guided by the Barcelona Clinic Liver Cancer (BCLC) staging system, which considers tumor size, liver function, and patient performance status [3].

Surgical resection remains the standard of care for early-stage HCC, with five-year overall survival (OS) rates ranging from 50% to 70% [4,5]. However, only about 10%-30% of patients are eligible for surgery due to tumor location or underlying liver dysfunction. For tumors ≤3 cm, thermal ablation techniques like radiofrequency ablation and microwave ablation achieve comparable efficacy to surgery, with local control rates of 70%-90% [6,7]. Transarterial chemoembolization (TACE) is commonly used in intermediate-stage HCC, whereas three-year OS rates remain low at 40% [8].

Stereotactic body radiation therapy (SBRT) represents a promising alternative for both operable and inoperable HCC. Unlike conventional radiation, SBRT uses image-guided techniques to deliver high-dose radiation in fewer fractions (typically 3-5 sessions), minimizing damage to healthy liver tissue. Studies show that SBRT achieves local control rates of 84%-92% at 1-2 years for tumors ≤5 cm, with three-year OS rates exceeding 70% [9-11]. The CyberKnife® system, which uses Synchrony® motion tracking, further enhances precision by synchronizing radiation delivery with tumor motion during respiration. However, challenges remain for tumors near the diaphragm [12]. Recent advancements have focused on markerless respiratory tracking systems, such as the Diaphragm Tracking (X-Sight) feature on CyberKnife, which uses infrared markers to model diaphragmatic motion [13]. This technique allows for non-invasive, real-time tracking with sub-millimeter accuracy, reducing the need for invasive marker placement.

We report one initial case of integrating diaphragm motion substitute tracking (DMST) into the CyberKnife respiratory model for SBRT treatment of diaphragm-adjacent HCC. This approach dynamically updates the respiratory model using orthogonal KV imaging and surface markers, pausing treatment if tracking error exceeds 5 mm. Preliminary results align with existing literature, highlighting the potential of this technique to expand the therapeutic window for challenging HCC locations.

## Case presentation

A 64-year-old male patient with a 30-year history of hepatitis B initially presented with dull right upper abdominal pain on August 26, 2017. A CT scan at a local hospital revealed a space-occupying lesion in the right hepatic lobe and liver cirrhosis. Subsequent evaluation via liver MRI (plain + contrast) at our center in August 2017 confirmed a suspicious HCC lesion in the upper segment of the right anterior hepatic lobe (2.2 × 2.1 cm), accompanied by mildly enlarged lymph nodes in the hepatic hilum and retroperitoneum, and an elevated AFP level of 18.15 ng/ml. He was diagnosed with primary hepatocellular carcinoma (cT2N0M0, BCLC-B, Child-Pugh A). From August 30 to September 5, 2017, he underwent CyberKnife SBRT targeting the hepatic lesion (the case received 7.5 Gy per fraction, 6 fractions, total dose of 45 Gy delivered every day to the PTV), followed by oral sorafenib and regular imaging follow-ups. By October 2017, contrast-enhanced MRI showed lesion shrinkage to 2.2 × 2.1 cm, which further decreased to 0.8 cm by May 2018. The lesion remained stable until July 2019, when it enlarged to 2.8 cm in maximum diameter. Subsequently, he underwent four sessions of TACE on August 1, 2019, October 18, 2019, August 3, 2020, and November 30, 2020. In October 2020, bone ECT detected multiple bone metastases, prompting a switch to oral lenvatinib. A follow-up MRI on November 29, 2020, revealed progressive disease with an enlarged active lesion in the right anterior hepatic lobe, cirrhosis, splenomegaly, mild ascites, and multiple enlarged lymph nodes in the hepatic hilum, retroperitoneum, and right cardiophrenic angle. Despite the interventions, the patient succumbed to HCC recurrence with widespread metastases in June 2021.

**Figure 1:**
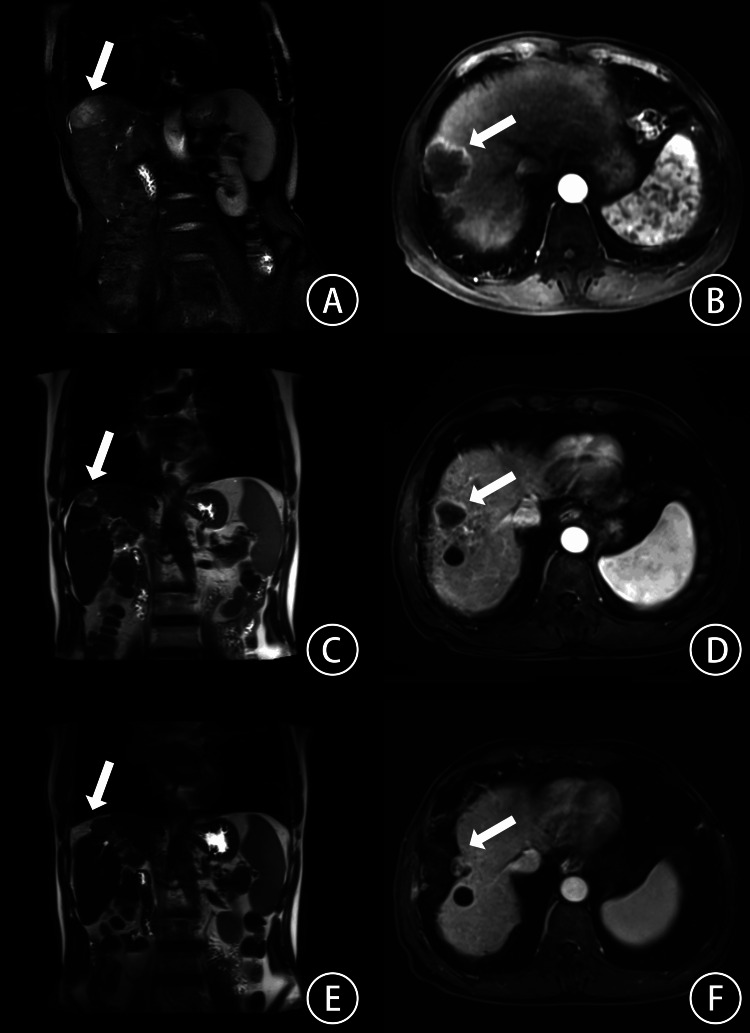
The MRI scan before and after SBRT A, C, and E. Coronal plane scanning; B, D, and F. Cross-sectional scanning. A and B. MRI scan before SBRT, C and D. MRI scan two months after SBRT, and E and F. MRI scan 17 months after SBRT. MRI, magnetic resonance imaging; SBRT, stereotactic body radiation therapy.

**Figure 2:**
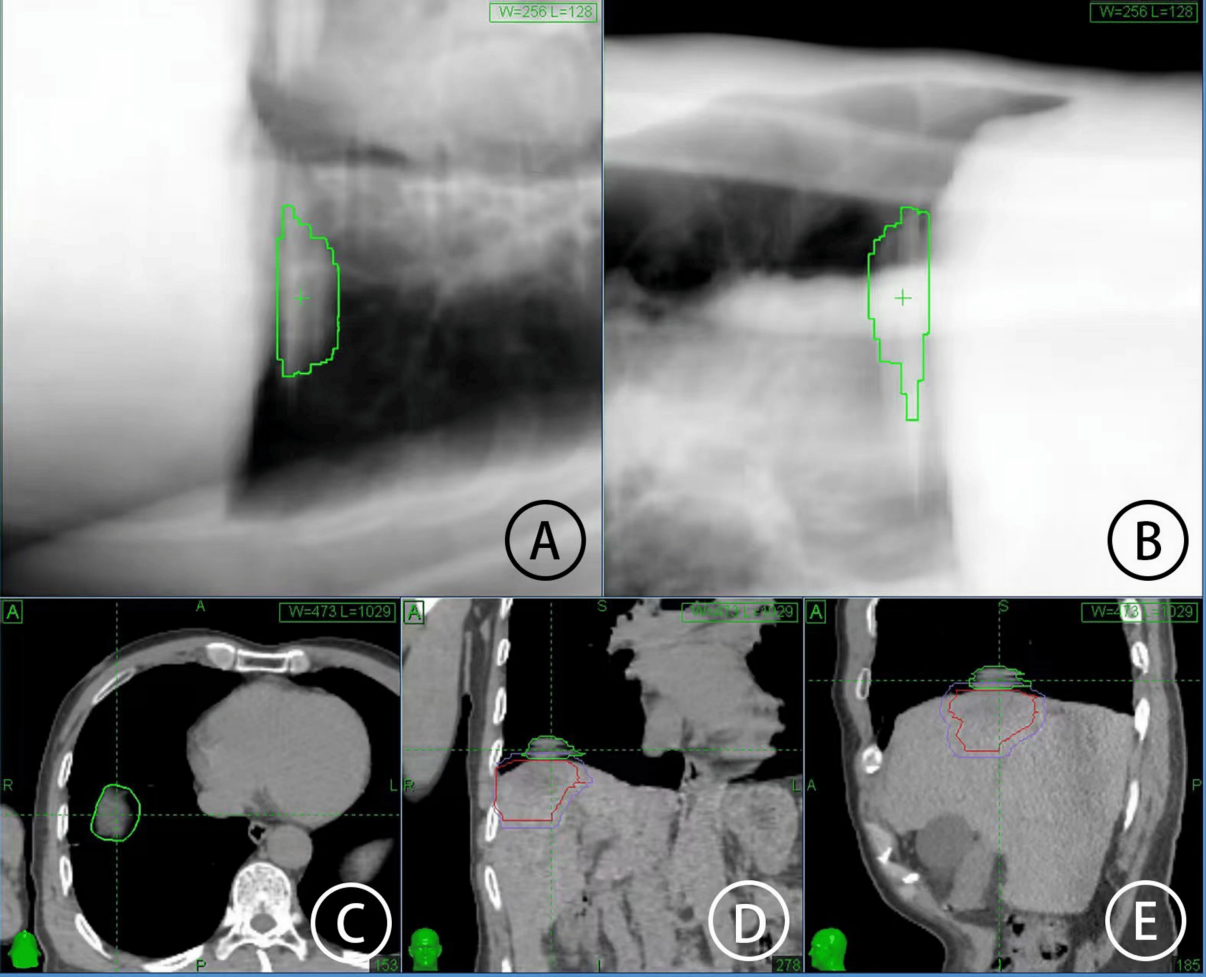
Real-time diaphragm tracking-guided imaging in plan simulation A and B. Diaphragmatic tracking zone in the simulation plane, C. Cross-sectional plane, D. Coronal plane, and E. Sagittal plane.

**Figure 3:**
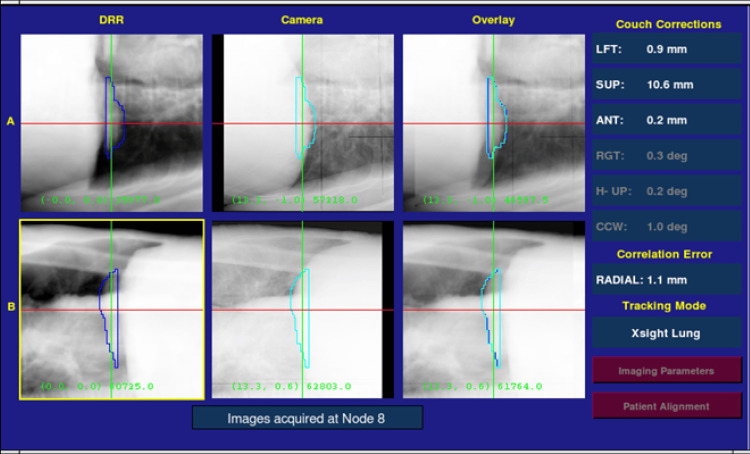
The print screen of the treatment delivery

The patient underwent SBRT using DMST with a dose of 45 Gy delivered in six fractions to the lesion, corresponding to a biologically effective dose (BED₁₀) of 78.75 Gy, and the mean treatment time per fraction was 48 minutes. The tumor diameter was 5 cm; post-treatment MRI showed that the tumor had shrunk to 0.8 cm. In this case, the patient had a local progression at 23 months and died at 46 months after SBRT. No grade 3 toxicities were observed in this patient (Figures 1-3).

SBRT was delivered on CyberKnife with a synchrony respiratory tracking system. Patients were placed in the supine position by using a whole-body vacuum pad. The normal organs at risk included the lung, heart, esophagus, stomach, liver-GTV, spinal cord, and kidney. The gross tumor volume (GTV) is defined as the visible tumor based on contrast-enhanced CT acquired on the portal-venous phase. The clinical target volume (CTV) is equal to the GTV. PTV was defined as the region of 5 mm outside of CTV.

The planning CT scan and enhanced portal-venous phase CT should be acquired under breath-free and breath hold (preferably end-expiratory), respectively. The scan range includes the whole liver, at least 10 cm above and below the tumor. The spiral thin-slice CT was acquired with 1.5 mm slice collimation and images were reconstructed in a slice thickness of 1.5 mm at the most. Co-registration of planning CT to contrast-enhanced CT is based on anatomical (spinal) fusion.

## Discussion

The recent meta-analyses have confirmed the positive role of SBRT in the treatment of liver cancer. A retrospective study focusing on smaller hepatocellular carcinomas reported a one-year local control (LC) rate of approximately 96% and a three-year LC exceeding 91% [14]. Another non-selective retrospective meta-analysis, including tumors of any size and stage, showed three- and five-year LC rates of 84% and 82%, respectively [15].

Liver tumor motion is significantly influenced by respiration, with the largest motion occurring in the superior-inferior (SI) direction, ranging from 5 to 50 mm [16-18]. Respiratory-induced organ motion poses a significant challenge to radiation therapy, potentially leading to target under-dosing and surrounding normal tissue over-dosing. To mitigate these uncertainties, techniques such as increasing safety margins, employing active/passive breath-hold, or implementing real-time target tracking have been adopted. Traditionally, fiducial markers are implanted near tumors to serve as surrogate tracking targets in X-ray images during SBRT, enabling real-time motion compensation. However, this invasive approach carries risks such as pneumothorax and marker migration, particularly for tumors adjacent to critical anatomical structures. Early animal studies indicated that liver motion magnitude correlates with the distance between the diaphragm and the measurement point. As an alternative, non-invasive techniques leverage the anatomical landmarks of the diaphragm dome to extract tumor motion information. A study by Yang et al. [19] showed that liver tumor motion had high concordance with diaphragm motion in SI and anterior-posterior directions, with closer tumor proximity to the diaphragm correlating with improved synchronization [20-23]. This observation supports the use of diaphragm motion as a reliable surrogate for tracking liver tumors, obviating the need for fiducial implantation.

Liver tumors located at the dome of the diaphragm are usually difficult to implant with metal markers, which can increase the risk of pneumothorax and bleeding. Using spine tracking requires delineating the internal target volume of tumor motion based on 4DCT, which can lead to more normal liver and lung tissues receiving excessive radiation. Considering that the respiratory motion model of diaphragmatic tumors is consistent with that of the diaphragm, we innovatively refined the X-Sight Lung technology to develop the DMST protocol. The brief steps are as follows: (i) Delineate the TTV (Tracking Target Volume), which involves contouring the liver region at the diaphragmatic dome; (ii) Use the TTV as the tracking region for XSight-Lung to develop the treatment plan; (iii) Treatment implementation: Utilizing a surface-synchronized camera to acquire the patient's respiratory waveform and simultaneously, employing an orthogonal kV imaging system to establish a respiratory-synchronized model targeting the TTV. This model is used for real-time tracking and model correction during the treatment process.

During clinical implementation, we established a rigorous quality control protocol: Treatment is manually paused by the technician when real-time tracking reveals a translational error exceeding 10 mm or a rotational error surpassing 2°, triggering an imaging verification process. If the translational or rotational errors remain non-compliant after imaging verification, the current respiratory model is deemed invalid, necessitating the reacquisition of respiratory signals and the reconstruction of a new motion model to ensure subsequent treatment accuracy. This mechanism ensures treatment safety through two tiers: real-time dynamic monitoring (displacement deviations between predicted and actual positions are compared every 60 seconds) and tiered response strategy (initial exceedance only requires imaging verification, while repeated exceedances prompt model reconstruction). 

## Conclusions

SBRT has demonstrated obviously improvements in local tumor control for liver cancer. In this case, we demonstrated how diaphragm-tracking SBRT was implemented for a hepatic lesion adjacent to the diaphragm, achieving local control outcome.
